# A Novel Interaction between MFN2/Marf and MARK4/PAR-1 Is Implicated in Synaptic Defects and Mitochondrial Dysfunction

**DOI:** 10.1523/ENEURO.0409-22.2023

**Published:** 2023-08-14

**Authors:** Yeongmi Cheon, Sunggyu Yoon, Jae-Hyuk Lee, Kiyoung Kim, Hyung-Jun Kim, Sung Wook Hong, Ye-Rang Yun, Jiwon Shim, Sung-Hak Kim, Bingwei Lu, Mihye Lee, Seongsoo Lee

**Affiliations:** 1 Gwangju Center, Korea Basic Science Institute, Gwangju 61751, Korea; 2 Laboratory of Molecular Biochemistry, Chonnam National University, Gwangju 61186, Korea; 3 Department of Microbiology and Molecular Biology, Chungnam National University, Daejeon 34134, Korea; 4 Department of Life Science, College of Natural Science, Hanyang University, Seoul 04763, Korea; 5 Department of Medical Science, Soonchunhyang University, Asan 31538, Korea; 6 Dementia Research Group, Korea Brain Research Institute, Daegu 41068, Korea; 7 Kimchi Functionality Research Group, World Institute of Kimchi, Gwangju 61755, Korea; 8 Department of Pathology, Stanford University School of Medicine, Stanford, California 94305; 9 Soonchunhyang Institute of Medi-Bio Science, Soonchunhyang University, Cheonan 31151, Korea

**Keywords:** *Drosophila melanogaster*, MARK4/PAR-1, MFN2/Marf, mitochondrial dynamics, neurodegenerative disease

## Abstract

As cellular energy powerhouses, mitochondria undergo constant fission and fusion to maintain functional homeostasis. The conserved dynamin-like GTPase, Mitofusin2 (MFN2)/mitochondrial assembly regulatory factor (Marf), plays a role in mitochondrial fusion, mutations of which are implicated in age-related human diseases, including several neurodegenerative disorders. However, the regulation of MFN2/Marf-mediated mitochondrial fusion, as well as the pathologic mechanism of neurodegeneration, is not clearly understood. Here, we identified a novel interaction between MFN2/Marf and microtubule affinity-regulating kinase 4 (MARK4)/PAR-1. In the *Drosophila* larval neuromuscular junction, muscle-specific overexpression of MFN2/Marf decreased the number of synaptic boutons, and the loss of MARK4/PAR-1 alleviated the synaptic defects of MFN2/Marf overexpression. Downregulation of MARK4/PAR-1 rescued the mitochondrial hyperfusion phenotype caused by MFN2/Marf overexpression in the *Drosophila* muscles as well as in the cultured cells. In addition, knockdown of MARK4/PAR-1 rescued the respiratory dysfunction of mitochondria induced by MFN2/Marf overexpression in mammalian cells. Together, our results indicate that the interaction between MFN2/Marf and MARK4/PAR-1 is fine-tuned to maintain synaptic integrity and mitochondrial homeostasis, and its dysregulation may be implicated in neurologic pathogenesis.

## Visual Abstract

**Figure:**
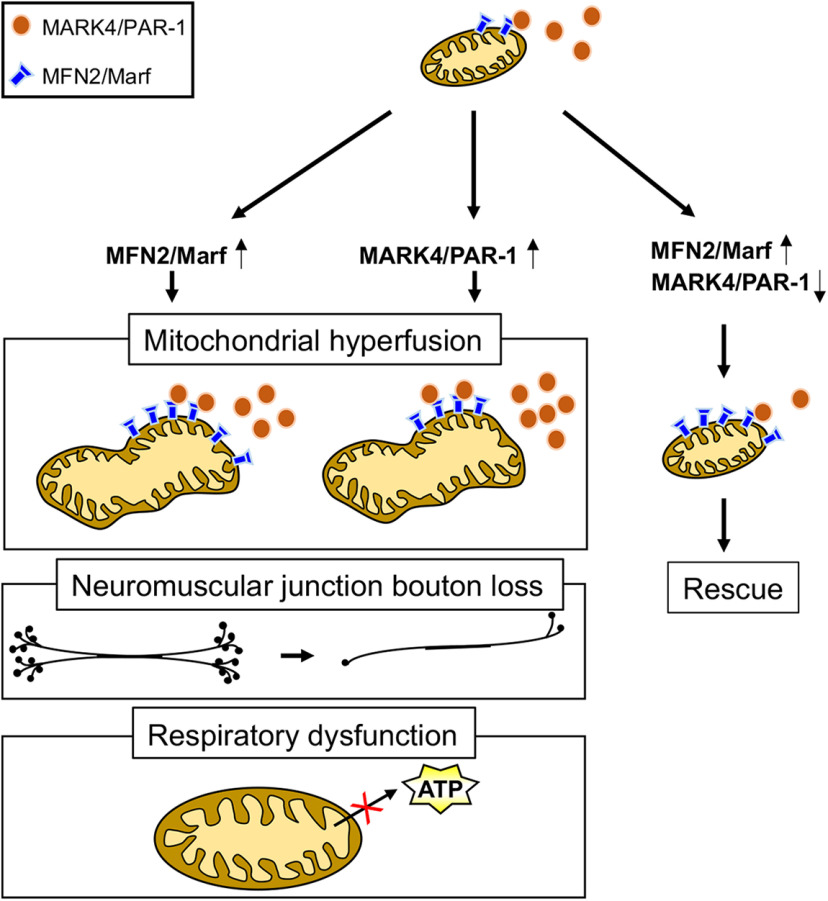

## Significance Statement

We identified a novel interaction between MFN2/Marf and kinase MARK4/PAR-1 in *Drosophila* and mammalian cells. The MFN2/Marf and MARK4/PAR-1 interaction was critical for maintaining the synaptic structure of neuromuscular junctions in *Drosophila*. In addition, we found that concomitant knockdown of MARK4/PAR-1 could rescue the mitochondrial hyperfusion and aberrant respiratory function caused by MFN2/Marf overexpression. Our study provides new insights into the link between mitochondrial defects and neurodegeneration, which makes a significant contribution to the understanding of neurologic pathogenesis and therapeutic development.

## Introduction

Mitochondria function to generate ATP through respiration and play a critical role in diverse cellular processes, including cellular metabolism, apoptosis, and calcium signaling (Sheridan and Martin, 2010; O'Rourke, 2016). The functional homeostasis of mitochondria is tightly coupled to their structural plasticity, for which they continuously change their size and interconnectivity (Mishra et al., 2014; Lee et al., 2017). The variability of mitochondrial morphology, termed mitochondrial dynamics, involves two main mechanisms—fusion and fission. Mitochondria form a network and undergo constant fusion and fission, which is required for adaptation to environmental stimuli and response to cytosolic signals. In addition, fusion and fission serve as quality control mechanisms. Impaired mitochondria are removed through fission and subsequent mitophagy, and mitochondria with normal function maintain quality by undergoing fusion. Fusion of mitochondria is mediated by several dynamin-related GTPases such as Mitofusin 1 (MFN1), Mitofusin 2 (MFN2), and Optic atrophy protein 1 (Opa1; Tilokani et al., 2018). In mouse embryonic fibroblasts, lack of *MFN1*, *MFN2*, or *Opa1* leads to aberrant mitochondrial morphology because of fragmentation of the network (Chen et al., 2003; Bocca et al., 2018). Mitochondrial fission is mediated by Dynamin-related protein 1 (Drp1), which interacts with mitochondrial receptor proteins (outer mitochondria membrane partners). Mitochondria in *Drp1*-deficient cells display a hyperelongated configuration with a highly connected network. Furthermore, dysregulation of mitochondrial membrane dynamics is linked to human diseases, and mutations in the *MFN2*, *Opa1*, and *Drp1* genes have been reported to cause several genetic disorders (Corrado et al., 2012).

Missense mutations of *MFN2* cause Charcot-Marie-Tooth disease type 2A with the pathogenic phenotype of peripheral axon degeneration (Iapadre et al., 2018). Loss of MFN2 in mice leads to lethality because of an early embryonic defect, and the conditional inactivation of MFN2 in the mouse hippocampus and cortex leads to the progression of neurodegeneration caused by mitochondrial morphologic changes (Chen et al., 2003; Jiang et al., 2018). In particular, overexpression of the mutant form of *MFN2* in adult mice induces progressive neurodegeneration and significant abnormalities in behavior, learning, and memory, which are similar to those seen in human neurodegenerative diseases (Ishikawa et al., 2019). Moreover, several studies have provided evidence implicating MFN2 in Alzheimer’s disease and amyotrophic lateral sclerosis (ALS). β-Amyloid (Aβ) protein was decreased in MFN2-impaired cells, indicating that the regulation of MFN2 might be associated with Alzheimer’s disease (Leal et al., 2016). In *Drosophila*, overexpression of mitochondrial assembly regulatory factor (Marf), a *Drosophila* ortholog of mammalian mitofusins, alleviated neuropathological defects induced by TDP-43 in a *Drosophila* ALS model (Khalil et al., 2017). Although it has been controversial whether loss of MFN2 induces or relieves the symptoms of neurodegenerative diseases, it is plausible that MFN2 activity needs to be fine-tuned to maintain proper neurobiological homeostasis. However, the regulation of MFN2/Marf, which underlies the neurodegeneration phenotype, remains largely unsolved (Jiang et al., 2018; Ishikawa et al., 2019).

Microtubule affinity-regulating kinase 4 (MARK4) is also a well-known gene linked to neurodegenerative disease. As a conserved serine/threonine kinase, MARK4 phosphorylates microtubule-associated proteins and modulates microtubule dynamics, and its role has been established in cell division control and cell polarity determination based on several studies in mammalian cells and *Drosophila* tissue (Naz et al., 2013; Wu and Griffin, 2017). MARK4 has also been reported to be involved in neuropathology. MARK4 overexpression hyperphosphorylates the microtubule-associated protein Tau, which causes its detachment from microtubules and eventually develops neurofibrillary tangles, a pathogenic hallmark of Alzheimer’s disease (Jenkins and Johnson, 2000; Trinczek et al., 2004; Lee et al., 2012). PAR-1, the *Drosophila* homolog of MARK4, phosphorylates a scaffold protein, Disk large (Dlg), which recruits diverse synaptic proteins. Overexpression of PAR-1 in muscle leads to hyperphosphorylation and disorganization of Dlg proteins and subsequently affects the degeneration of synaptic boutons in larval muscle (Zhang et al., 2007). PAR-1 also phosphorylates Tau at the S262 and S356 residues, as its homolog MARK4 does, which is implicated in Alzheimer’s disease-related tau toxicity (Nishimura et al., 2004; Ando et al., 2016).

Here, we discovered that the postsynaptic overexpression of Marf in muscle dramatically reduces the synaptic bouton number of neuromuscular junctions (NMJs) in *Drosophila* larvae. The defect in synaptic morphology was rescued by knockdown of PAR-1. We also found a physical association between MFN2/Marf and MARK4/PAR-1. The role of the interaction of MFN2/Marf and MARK4/PAR-1 in mitochondrial dynamics and function was also verified. MARK4/PAR-1 knockdown alleviates hyperfusion phenotypes of mitochondria and aberrant respiratory capacity induced by MFN2/Marf overexpression. Hence, our results suggest that the interaction between MFN2/Marf and MARK4/PAR-1 has an effect on mitochondrial homeostasis and peripheral neuron maintenance.

## Materials and Methods

### Drosophila *strains*

The *UAS*-*PAR-1* and *PAR-1^W3^* mutant lines have been described previously (Tomancak et al., 2000; Lee et al., 2012). *Mhc*-*GAL4* was provided by J Troy Littleton. *PAR-1* RNAi, *Marf* RNAi, *Opa1* RNAi, *Drp1* RNAi, *UAS*-*hMFN1*, *UAS*-*hMFN2*, and *UAS-mito-GFP* lines were obtained from the Bloomington Drosophila Stock Center. The following transgenic stocks are available from Korea Drosophila Resource Center: *UAS*-*Marf*-*FLAG* (stock #10145), *UAS*-*Opa1*-*FLAG* (stock #10144), and *UAS*-*Drp1* (stock #10142). Flies were maintained at 25°C on a standard *Drosophila* medium.

### Neuromuscular junction staining

To stain the larval neuromuscular junction, both males and females were collected. The larvae were dissected in PBS and fixed in 4% paraformaldehyde (PFA) in PBS for 20 min. After fixation, the sample was collected in a 0.7 ml microcentrifuge tube in 0.1% Triton X-100 in PBS (PBS-T) and washed three times in 0.1% PBS-T every 5 min. FITC-conjugated anti-HRP (1:150; Jackson ImmunoResearch) was added, and the sample was incubated for 2 h at room temperature. Samples were mounted using the SlowFade Antifade kit (Invitrogen).

Confocal images were collected from a Leica TCS SP5 AOBS (Acousto Optical Bream Splitter) confocal microscope equipped with a 40× inverted NX oil lens, located at Gwangju Center, Korea Basic Science Institute. To analyze the neuromuscular junction, we used the procedure described previously (Lee et al., 2012).

### Cell culture, transfection, and RNA interference

The mammalian cell lines 293T, Neuro-2a (CCL-131, ATCC) and C2C12 (a gift from Tae Hyun Youm, CRL-1772, ATCC; Youm et al., 2019) were maintained in DMEM containing 10% fetal bovine serum and 1% penicillin/streptomycin (Invitrogen). Cells were transfected with either Lipofectamine 3000 (Thermo Fisher Scientific) or Effectene (Qiagen) according to instructions from the manufacturer. Small interfering RNAs (siRNAs) were synthesized by Bioneer and targeted to the following sequences: mouse *MARK4*, 5′-GGGATCTAAAGGCTGAAAA-3′ (#1), 5′-GGTCGCTATTAAGATCATT-3′ (#2), and 5′-GGCCAACATCAAAATCGCC-3′ (#3; Kuhns et al., 2013).

### Immunoblotting and protein binding assay

For immunoprecipitation, the cells were harvested and lysed in binding buffer (50 mm Tris-Cl, pH 8.0, 150 mm NaCl, 5 mm EDTA, 5 mm EGTA, 10% glycerol, 10 mm NaF, and 0.1% NP-40), supplemented with protease (Roche) and phosphatase inhibitor cocktails (Thermo Fisher Scientific). The lysates were incubated with 1 μg of the appropriate antibodies and Protein A Sepharose beads (GE Healthcare) for 4 h at 4°C. The following antibodies were used for immunoblot analysis: mouse anti-MFN2 (1:1000; Abcam) and mouse anti-VDAC1 (1:1000; Abcam), rabbit anti-MARK4 (1:1000; Cell Signaling Technology) and rabbit anti-MYC (1:2000; Cell Signaling Technology), and mouse anti-FLAG (1:2000; Millipore) and mouse anti-α-tubulin (1:5000; Millipore). Anti-Marf antiserum was obtained by immunizing rats with recombinant Marf peptide, DTVDKSGPGSPLSRF (Ziviani et al., 2010).

### Expression plasmids

The *MFN2-YFP* plasmid (catalog #28010) was purchased from Addgene. Full-length and truncated mutants (encoding aa 1–600, 1–350, and 601–757) of human *MFN2* (NM_014874) were subcloned into a pcDNA3.1-3FLAG mammalian expression vector (Invitrogen) at the BamHI and EcoRI sites. The MYC-tagged full-length PAR-1 construct was described previously (Lee et al., 2012). The human MARK4-MYC construct was provided by Kanae Ando (Saito et al., 2019). To visualize mitochondria in mammalian cells, the following plasmids were used: pDsRed2-Mito construct obtained from Clontech and pLV-mitoGFP construct provided by Pantelis Tsoulfas (plasmid #44385, Addgene; Kitay et al., 2013). The GFP-ER (endoplasmic reticulum) plasmid was a gift from Soojin Lee (Chungnam National University), which involves N-terminal ER signal sequence and C-terminal ER retention signal (KDEL).

### *Drosophila* larval muscle and adult wing muscle immunohistochemistry

To stain the mitochondria in larval muscle, both males and females were collected at wandering third instar larvae, dissected in PBS, and fixed in 4% PFA in PBS for 20 min. After fixation, the sample was placed in a 0.7 ml tube in 0.1% PBS-T and washed three times in 0.1% PBS-T every 10 min. Primary antibody staining with mouse anti-ATP5α (1:1000; Abcam) was performed at 4°C overnight. The samples were washed and then incubated in FITC-conjugated anti-mouse antibody (1:250; Jackson ImmunoResearch) for 3 h at room temperature. Samples were mounted using the SlowFade Antifade kit (Invitrogen).

To stain the mitochondria in adult wing muscle, adult female flies (5 d after eclosion) were selected. The thoraces were dissected in PBS and then collected in a 0.7 ml tube containing ice-cold PBS. Afterward, the samples were fixed in 4% PFA in PBS for 30 min. After fixation, the samples were washed three times with 0.1% PBS-T every 10 min. Primary antibody staining with anti-ATP5α (1:1000; Abcam) was performed at 4°C overnight. The sample was incubated with FITC-conjugated anti-mouse secondary antibody (1:250; Jackson ImmunoResearch) at 4°C overnight. Samples were mounted using the SlowFade Antifade kit (Invitrogen).

Confocal images were collected using a Leica TCS SP5 AOBS confocal microscope equipped with a 63× inverted NX oil lens, located at Gwangju Center, Korea Basic Science Institute.

### Quantification of mitochondria size in adult wing muscle

*Drosophila* mitochondrial morphology was quantified using the revised protocol of a previous study (Wang et al., 2016). Each image of adult wing muscle was processed using ImageJ software (http://imagej.nih.gov/ij/). The binary image could be obtained by the threshold tool, which ranges from 70 to 75%, and modified manually to separate the mitochondria. Next, we selected each mitochondrion using the wand (tracing) tool and calculated the number of pixels using the measurement tool. Using Microsoft Excel, the pixel value was converted to the actual size value (the pixel area was 0.0039 μm^2^).

### Mitochondrial fractionation in *Drosophila* muscle tissue

Mitochondrial fractionation of *Drosophila* muscle tissue was performed using the Mitochondria Isolation Kit for Tissue (Thermo Fisher Scientific). Both males and females at wandering third-instar larvae were used for muscle extraction. After the samples were collected, the muscles were homogenized in 200 μl bovine serum albumin-containing reagent A per sample. Then, the 3 μl reagent B solution was added per sample and vortexed for 5 s. The tube was incubated on ice for 5 min and vortexed at maximum speed every minute. Reagent C, 200 μl, was added per sample, and the tube was gently inverted several times. The tube was centrifuged at 700 × *g* for 10 min at 4°C, and the supernatant was transferred to a new tube. Subsequently, the supernatant was centrifuged at 12000 × *g* for 15 min at 4°C, and the supernatant was removed. The supernatant was used as a cytosolic fraction sample. The pellet was resuspended in washing buffer, 500 μl per sample, and centrifuged at 12000 × *g* for 5 min at 4°C. The supernatant was removed, and the pellet was collected as the mitochondrial fraction. Protein samples were denatured using 2× Laemmli Sample Buffer (Bio-Rad).

### Mitochondrial fractionation from mammalian cells

Preparation of a crude mitochondrial fraction from Neuro-2a cells was performed using a previously published method (Wieckowski et al., 2009). Briefly, the cells were harvested and carefully homogenized by 50–60 strokes with a glass pestle (Corning) on ice. All centrifugations were conducted at 4°C. Nuclei and unbroken cells were pelleted at 600 × *g* for 10 min, followed by centrifugation of the supernatant at 7000 × *g* for 15 min to isolate crude mitochondria from the cytosolic fraction. To remove the remaining contaminants (e.g., microsomes and plasma membranes), the collected mitochondrial pellet was resuspended and centrifuged at 10000 × *g* for 10 min. For subfractionation, the cytosolic fraction was centrifuged at 20000 × *g* for 30 min to obtain the pellet (plasma membrane). Further centrifugation of the collected supernatant at 100000 × *g* for 1 h resulted in the isolation of cytoplasm and ER (pellet). The collected fractions were used for immunoblot analysis.

### *In situ* proximity ligation assay and immunofluorescence microscopy

The proximity ligation assay (PLA) was performed on fixed Neuro-2a or C2C12 cells with Duolink PLA technology reagents (Sigma-Aldrich) following instructions from the manufacturer (Cheon et al., 2021). Briefly, cells cultured on 35 mm glass bottom dishes (SPL Life Sciences) were fixed with 4% paraformaldehyde in PBS for 15 min and permeabilized with 0.1% Triton X-100 in PBS for 10 min. After blocking for 1 h at 37°C, the cells were incubated with rabbit anti-MARK4 (Abcam) and mouse anti-MFN2 (Abcam) antibodies for 2 h at room temperature, treated with a pair of fluorescently labeled oligonucleotide probes for 1 h, and incubated with ligase solution for 30 min at 37°C. Next, the ligated oligonucleotide was amplified for 90 min at 37°C and mounted with a mounting solution containing DAPI.

For the colocalization analysis of GFP-positive subcellular organelles and PLA dots, the green and red fluorescence signals were captured simultaneously by using the 488 and 594 nm laser lines, respectively, under a confocal laser scanning microscope with 63×/1.4 oil objective (Zeiss LSM 880 with Airyscan, Zeiss). Next, the gray values were analyzed by the plot profile function in ImageJ software.

### Extracellular flux analysis

A Seahorse XFe96 Extracellular Flux Analyzer (Agilent) was used to measure the cellular oxygen consumption rate (OCR). Briefly, Neuro-2a cells transfected with *MFN2*-*FLAG* and/or *MARK4* siRNAs were plated onto a 96-well Seahorse microplate at 2 × 10^4^ cells/well and incubated at 37°C in a 5% CO_2_ incubator for 18 h. On the day of analysis, cells were washed twice and replaced with unbuffered DMEM medium containing the following (in mm): 25 glucose, 1 sodium pyruvate, and 2 glutamine, followed by incubation at 37°C in a non-CO_2_ incubator for 45 min. After measuring OCR baseline, the cells were treated with a sequential injection of mitochondrial inhibitors containing the following (in mm): 1.5 oligomycin, 0.5 FCCP, and 0.5 rotenone/antimycin A. Total protein concentration was quantified by BCA Assay (Thermo Fisher Scientific) and normalized for each well.

### Statistical analysis

Statistical analysis was performed using GraphPad Prism software, version 8. Comparisons of different groups in the mean values of multiple datasets were performed using one-way ANOVA with Tukey’s multiple comparison test. All values are presented as the mean ± SD; *p < 0.05, **p < 0.01 and ***p < 0.001.

## Results

### Marf genetically interacts with PAR-1

**Figure 1.**
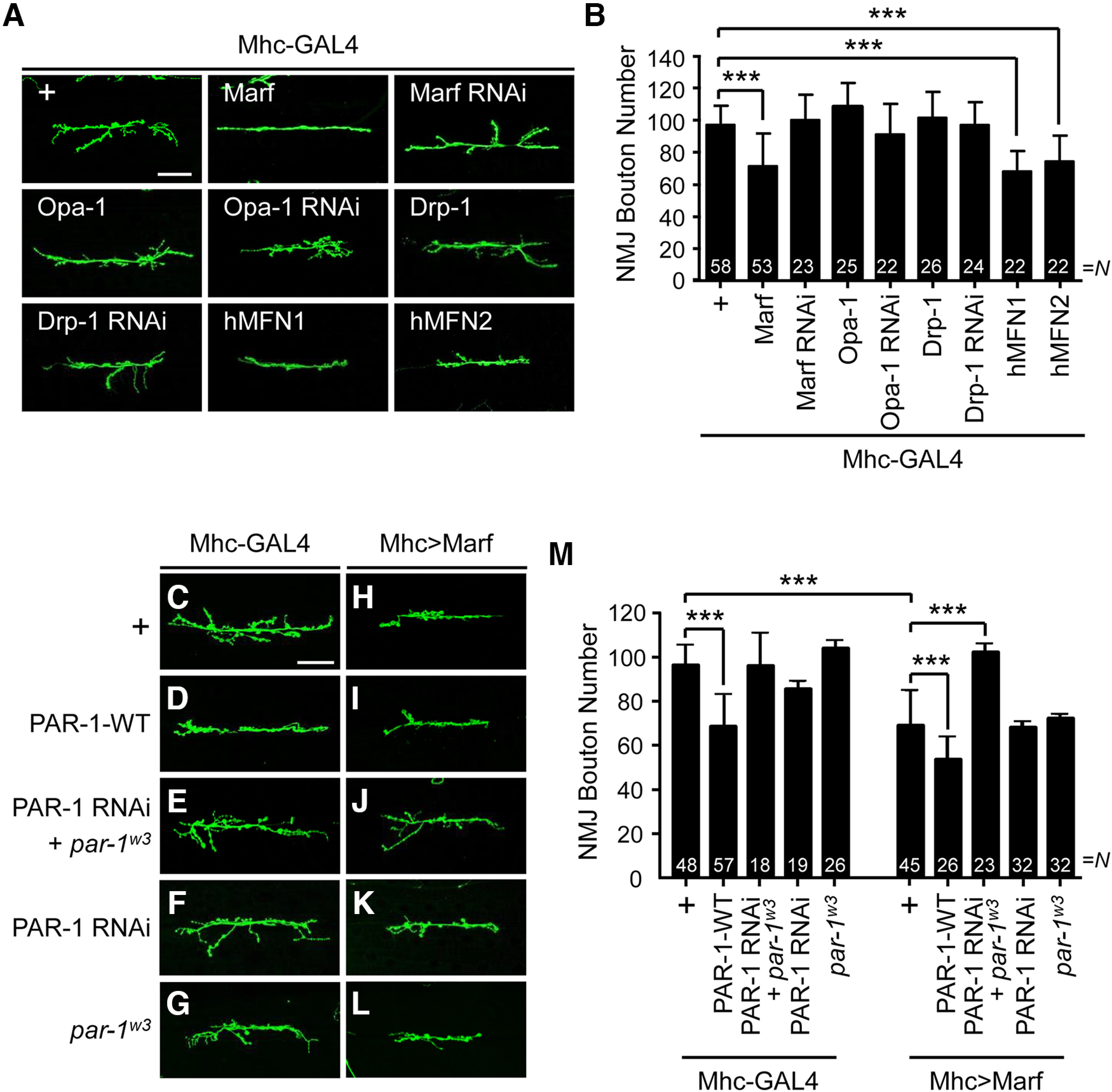
Genetic interaction between *Marf* and *PAR-1* in *Drosophila* larval NMJ. ***A***, Representative NMJ images of larval muscle 6/7 in segment A3 immunostained with an anti-HRP antibody. The genotypes are as follows: Mhc-GAL4/+ as control, Mhc-GAL4>UAS-Marf-FLAG, Mhc-GAL4>UAS-Marf RNAi, Mhc-GAL4>UAS-Opa-1-FLAG, Mhc-GAL4>UAS-Opa-1 RNAi, Mhc-GAL4>UAS-Drp-1-HA, Mhc-GAL4>UAS-Drp-1 RNAi, Mhc-GAL4>UAS-hMFN1, and Mhc-GAL4>UAS-hMFN2. Scale bar, 50 μm. ***B***, Quantification of data indicates that Marf, hMFN1, and hMFN2 overexpression reduced the total number of boutons. ***C–L***, Representative NMJ images of larval muscle 6/7 in segment A3 immunostained with an anti-HRP antibody. The genotypes are Mhc-GAL4/+ as control (***C***), Mhc-GAL4>UAS-PAR-1-WT (***D***), *par-1^w3^*+Mhc-GAL4>UAS-PAR-1-RNAi (***E***), Mhc-GAL4>UAS-PAR-1 RNAi (***F***), *par-1^w3^*+Mhc-GAL4/+ (***G***), Mhc-GAL4>UAS-Marf-FLAG (***H***), Mhc-GAL4>UAS-Marf-FLAG+UAS-PAR-1-WT (***I***), *par-1^w3^*+Mhc-GAL4>UAS-Marf-FLAG+UAS-PAR-1 RNAi (***J***), Mhc-GAL4>UAS-Marf-FLAG+UAS-PAR-1 RNAi (***K***), and *par-1^w3^*+Mhc-GAL4>UAS-Marf-FLAG (***L***). Scale bar, 50 μm. ***M***, Quantification of data shown in ***C–L***. The NMJ area was selected to quantify the number of boutons. *N* indicates the number of NMJs analyzed in each group. The data are shown as mean ± SD. Statistical significance was determined by a one-way ANOVA test; ***p < 0.001. The data that presynaptic manipulation of Marf didn’t change the NMJ bouton number are shown in Extended Data Figure 1-1.

To investigate the neurodegenerative effect of the proteins involved in *Drosophila* mitochondrial dynamics, we observed synaptic morphology at the larval NMJ after tissue-specific knockdown or overexpression of a target gene (Fig. 1*A*). We examined the NMJ synaptic bouton number in larval muscle 6/7 of the A3 segment (Lee et al., 2012) and found that postsynaptic overexpression of Marf induced a 28% reduction in the synaptic bouton number compared with the control (Fig. 1*A*,*B*,*H*,*M*). In addition, the ectopic expression of the human orthologues Mitofusin 1 and 2 (hMFN1, hMFN2) at postsynaptic compartment caused 30 and 22% loss of boutons, respectively (Fig. 1*A*,*B*). In contrast, the presynaptic expression of Marf did not affect the NMJ structure (Extended Data Fig. 1-1). In the postsynaptic compartment, the kinase PAR-1 plays a critical role in the regulation of synaptic structure (Zhang et al., 2007; Lee et al., 2012). To test whether Marf is functionally associated with PAR-1, we analyzed the effect of epistasis of *Marf* and *PAR-1* expressions on NMJ morphology. Similar to a previous study, postsynaptic overexpression of PAR-1 resulted in a reduction in bouton number (28%) compared with the control (Fig. 1*D*,*M*; Zhang et al., 2007). When Marf and PAR-1 were overexpressed simultaneously, loss of synaptic boutons was significantly enhanced, showing a 45% decrease compared with the control (Fig. 1*I*,*M*). Interestingly, PAR-1 knockdown in combination with *PAR-1* RNAi and *PAR-1* mutant, *par-1^w3^*, largely reduced Marf-induced synaptic abnormalities (Fig. 1*J*,*M*). Note that the expression of either *PAR-1* RNAi or *par-1^w3^* heterozygote alone was not sufficient (Fig. 1*K–M*). Collectively, we found that overexpression of Marf results in synaptic outgrowth, and knockdown of PAR-1 alleviated the synaptic defect caused by postsynaptic expression of Marf. These results suggest that Marf and PAR-1 genetically interact at the postsynaptic compartment to maintain the synaptic structure of NMJs.

10.1523/ENEURO.0409-22.2023.f1-1 Figure 1-1 Presynaptic-specific overexpression or downregulation of Marf does not affect NMJ bouton number. ***A***, Representative NMJ images of larval muscle 6/7 in segment A3 immunostained with an anti-HRP antibody. The genotypes are as follows: elav-GAL4/+ as control, elav-GAL4>UAS-Marf-FLAG, and elav-GAL4>UAS-Marf RNAi. Scale bar, 50 μm. ***B***, Quantification of data shows that Marf overexpression in presynapse has no effect on the number of boutons. The data are shown as mean ± SD. Statistical significance was determined by one-way ANOVA test; n.s. Download Figure 1-1, TIF file.

### MFN2/marf physically interacts with MARK4/PAR-1

**Figure 2.**
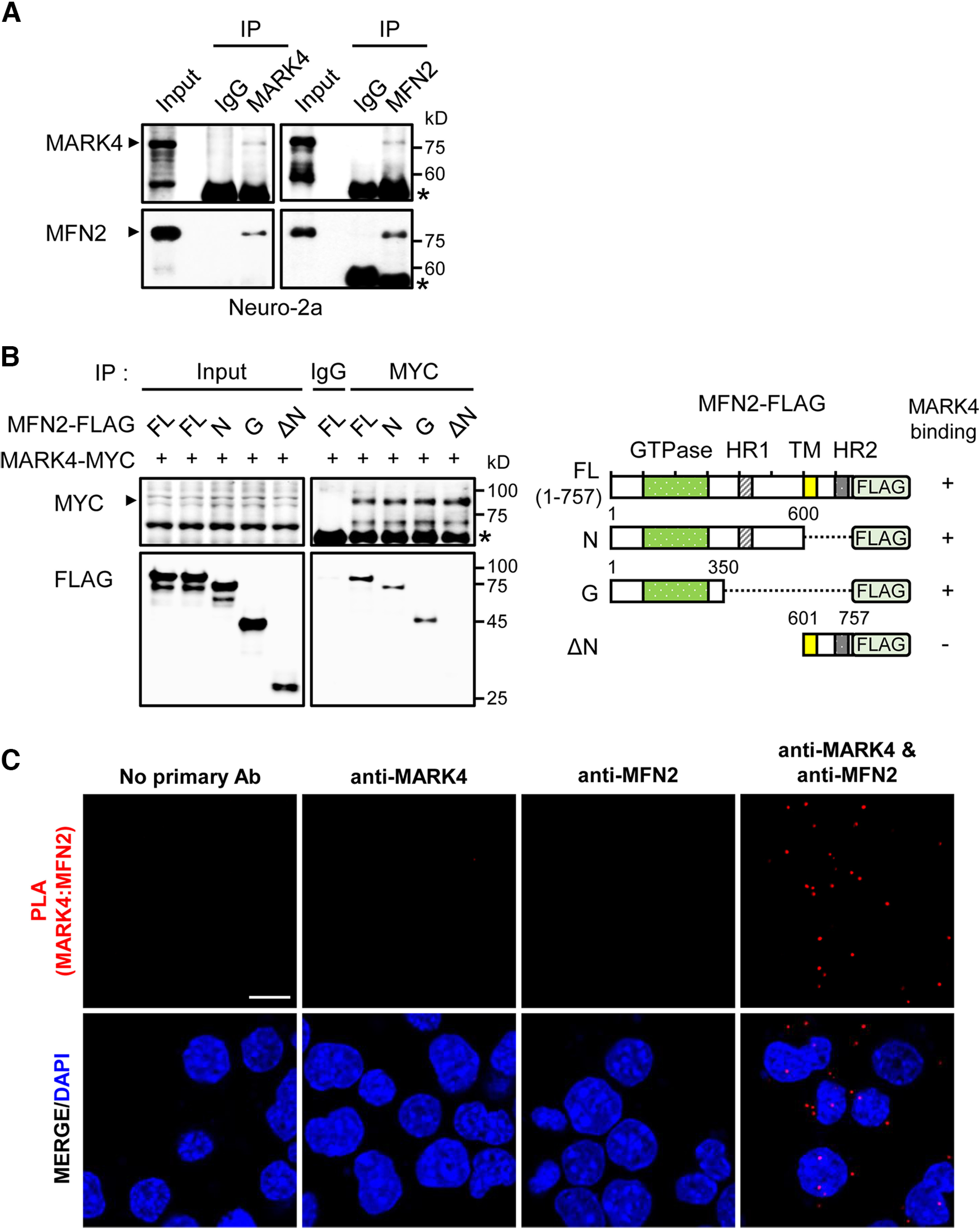
MFN2/Marf physically interacts with MARK4/PAR-1. ***A***, Interaction between endogenous MARK4 and MFN2. Neuro-2a cell lysates were subjected to IP with indicated antibodies. Asterisks indicate IgG bands. ***B***, Determination of MFN2 domain required for MARK4 binding. Various truncated mutants of FLAG-tagged MFN2 were constructed, and IP was performed after indicated constructs were transfected into 293T cells. An arrowhead marks MARK4-MYC. Asterisk indicates IgG band. GTPase, amino acid 103–307; Heptad repeat coiled-coil region 1 (HR1), amino acid 391–434; transmembrane domain (TM), amino acid 605–647; and HR2, amino acid 695–738. ***C***, Protein–protein interaction by *in situ* PLA. *In situ* PLA was performed on fixed Neuro-2a cells with Duolink PLA technology. After incubation with rabbit anti-MARK4 and/or mouse anti-MFN2 antibodies for 2 h, the cells were treated with PLA probes for 1 h and mounted with a mounting solution containing DAPI. Scale bar, 10 μm.

Next, we investigated possible physical association between MFN2/Marf and MARK4/PAR-1. To test physical association of these proteins, we conducted coimmunoprecipitation (co-IP) experiments with anti-MARK4 or anti-MFN2 antibodies using the mammalian cell line Neuro-2a. MFN2 was detected in the MARK4-enriched precipitate, and MARK4 was also coisolated with MFN2 (Fig. 2*A*).

To determine which domain of MFN2 is indispensable for MARK4 binding, we performed co-IP of 293T cell lysates expressing MYC-tagged MARK4 and FLAG-tagged MFN2 truncated constructs including full length (amino acid 1−757, N (amino acid 1−600), G (amino acid 1−350), and ΔN (amino acid 601−757; Fig. 2*B*). Like the full-length MFN2, the N and G but not ΔN constructs exhibited high affinity for MARK4-MYC (Fig. 2*B*). These data supported that the N-terminal region of MFN2 plays a critical role in the physical association between MFN2 and MARK4.

To further verify the interaction of MARK4 and MFN2 *in vivo*, we performed *in situ* PLA with anti-MARK4 and anti-MFN2 antibodies using Neuro-2a cells (Fig. 2*C*). Although no signals were observed in cells incubated with a single antibody, clear PLA signals were detected when both primary antibodies were present, indicating the close proximity of MARK4 and MFN2. These results provide strong support to explain the physical interaction of MARK4 and MFN2.

### MARK4/PAR-1 regulates mitochondrial fusion induced by MFN2/marf

**Figure 3.**
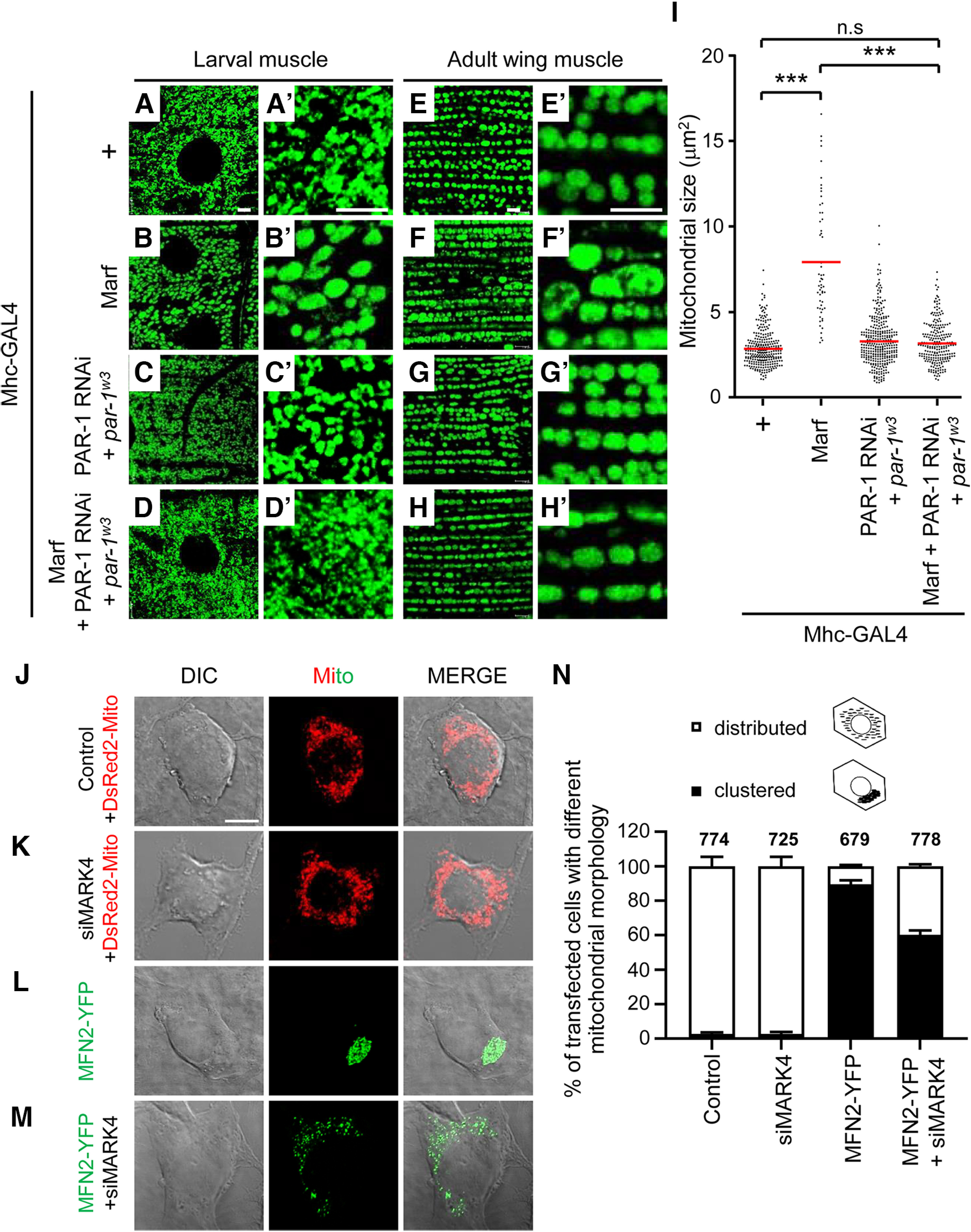
***A–H’***, MARK4/PAR-1 knockdown rescues the mitochondria hyperfusion caused by MFN2/Marf overexpression. The mitochondria in the larval muscle 6/7 of the segment A3 (***A–D***) and adult wing muscle (***E–H***) were immunostained with anti-ATP5α antibody. Each image is magnified on the right (***A’–H’***). Scale bar, 5 μm. ***I***, Quantification of mitochondrial size in the adult wing muscle. The number of mitochondria analyzed in each genotype are as follows: Mhc-GAL4/+ (*n* = 291), Mhc-GAL4>UAS-Marf-FLAG (*n* = 57), *par-1^w3^*+Mhc-GAL4>UAS-PAR-1-RNAi (*n* = 352), and *par-1^w3^*+Mhc-GAL4>UAS-Marf-FLAG+UAS-PAR-1-RNAi (*n* = 238). The data are shown as mean ± SD. Statistical significance was determined by a one-way ANOVA test; ***p < 0.001; n.s. indicates not significant. ***J–M***, Representative images of mitochondria in Neuro-2a cells expressing either DsRed2-Mito or MFN2-YFP along with MARK4 siRNA (100 nm). Mito-DsRed2 (red) was used to label mitochondria. Scale bar, 10 μm. ***N***, Quantification of the transfected cells with indicated mitochondrial morphology shown in ***J–M***. In each condition, ∼700 cells were scored, and mean ± SD was presented. White box, distributed mitochondria as a normal phenotype; black box, asymmetrical perinuclear clustering of mitochondria. The data supporting the overexpression of PAR-1 in larvae and adult muscle-induced hyperfused mitochondria is provided in Extended Data Figure 3-1, *A–G*. The data providing MFN2 colocalize with mitochondria in Neuro-2a cell is shown in Extended Data Figure 3-1*H*.

Considering the fact that MFN2/Marf mainly functions to fuse mitochondria, we tested whether MARK4/PAR-1 might be involved in the regulation of mitochondrial morphology based on its interaction with MFN2/Marf. To observe the morphology of mitochondria, we immunostained the larval A3 muscle or adult wing muscle with an anti-ATP5α antibody to label the mitochondrial complex (Chen et al., 2015). Muscle-specific Marf overexpression induced hyperfusion of mitochondria in larval muscle compared with the control (Fig. 3*A*,*B*). Furthermore, the mitochondria in adult wing muscle showed hyperelongated morphology with an approximately threefold increase in size compared with the control (Fig. 3*E*,*F*,*I*). Although the knockdown of PAR-1 alone had no significant effect on mitochondrial morphology (Fig. 3*C*,*G*; Extended Data Fig. 3-1), downregulation of PAR-1 surprisingly rescued the mitochondrial hyperfusion caused by Marf overexpression in larval and adult muscle (Fig. 3*D*,*H*,*I*). To further validate the role of MARK4 in MFN2-dependent mitochondrial shape change, we observed mitochondrial morphology in Neuro-2a cells transfected with either *DsRed2-Mito* or *MFN2-YFP* along with *MARK4* siRNA. In the control cells, normal tubular mitochondria marked by DsRed2-Mito were distributed evenly throughout the cytoplasm (>97% cells; Fig. 3*J*,*N*), and a similar mitochondrial phenotype was detected in *MARK4* siRNA-incubated cells (Fig. 3*K*,*N*). However, when overexpressed, MFN2 induced the formation of perinuclear mitochondrial clusters in ∼90% of the cells (Fig. 3*L*,*N*; Extended Data Fig. 3-1*H*); although not drastic, MARK4 depletion with siRNA partially suppressed the abnormal mitochondrial morphology caused by MFN2 overexpression in ∼30% of cells (Fig. 3*M*,*N*). These results suggest that MARK4/PAR-1 participates in mitochondrial dynamics through the regulation of MFN2/Marf.

10.1523/ENEURO.0409-22.2023.f3-1 Figure 3-1 Overexpression of PAR-1 in muscles results in mitochondrial hyperfusion. ***A–F***, The mitochondria were visualized in the larval muscle 6/7 of A3 by UAS-mito-GFP (***A–C***) and in adult wing muscle by immunostaining with anti-ATP5α antibody (***D–F***). Each image is magnified on the right (***A’–F’***). Scale bar, 5 μm. The genotypes of larval muscle are Mhc-GAL4>UAS-mito-GFP as a control (***A***), Mhc-GAL4>UAS-mito-GFP+UAS-PAR-1-WT (***B***), and Mhc-GAL4>UAS-mito-GFP+UAS-PAR-1 RNAi (***C***). The genotypes of adult muscle are Mhc-GAL4/+ as a control (***D***), Mhc-GAL4>UAS-PAR-1-WT (***E***), and Mhc-GAL4>UAS-PAR-1 RNAi (***F***). ***G***, Quantification of mitochondrial size in the adult wing muscle. The number of mitochondria analyzed in each genotype is as follows: Mhc-GAL4/+ (*n* = 133), Mhc-GAL4>UAS-PAR-1-WT (*n* = 145), and Mhc-GAL4>UAS-PAR-1 RNAi (*n* = 77). The data are shown as mean ± SD. Statistical significance was determined by a one-way ANOVA test; ***p < 0.001; n.s. ***H***, MFN2-YFP colocalizes with DsRed-Mito in a clustered pattern. Representative images of Neuro-2a cells expressing MFN2-YFP (green) and DsRed2-Mito (red). DAPI is shown in blue, and co-localized regions were indicated as yellow. Scale bar, 10 μm. Download Figure 3-1, TIF file.

### MARK4/PAR-1 is localized to the mitochondria and regulates mitochondrial function

**Figure 4.**
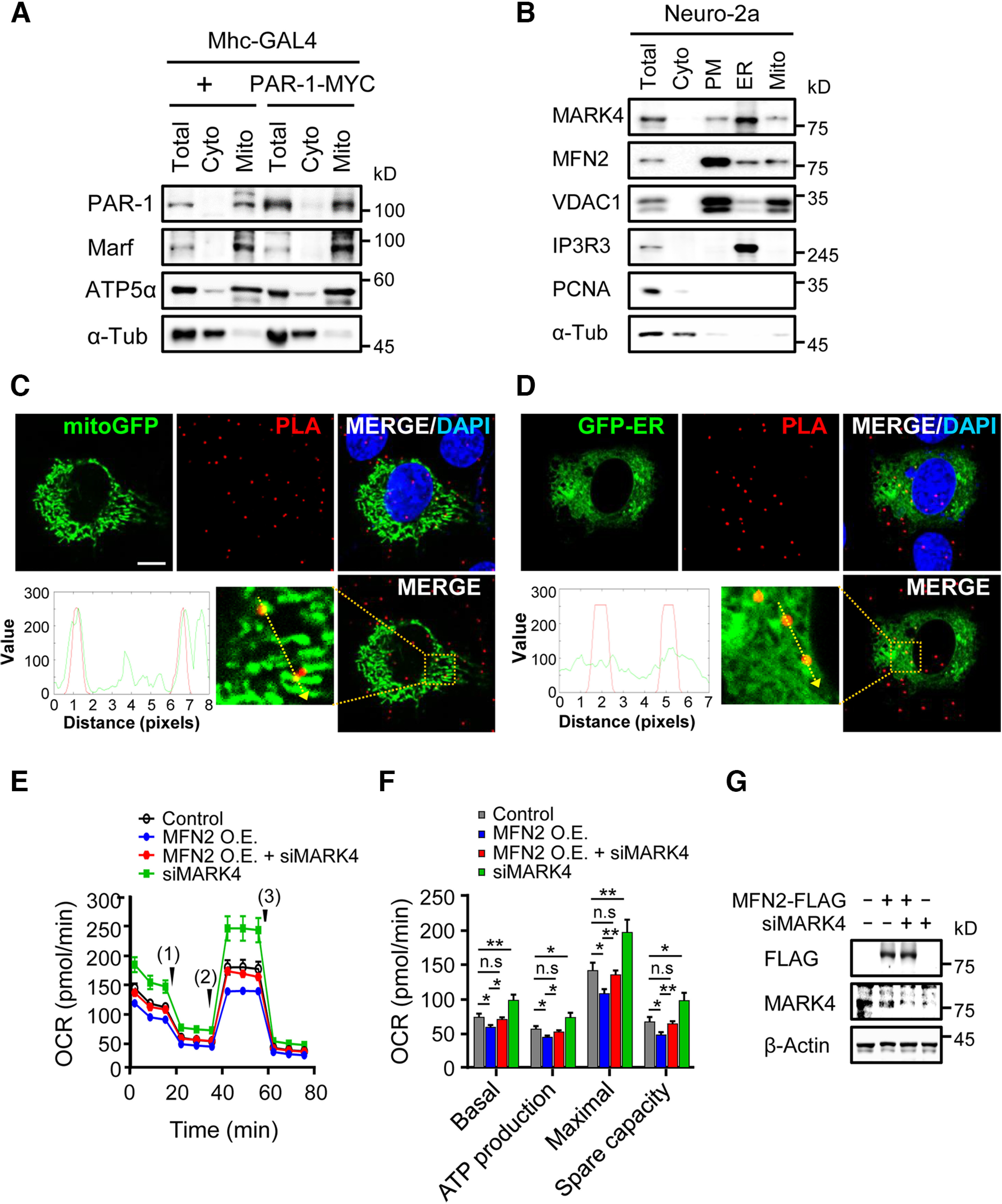
MARK4/PAR-1 localizes at the mitochondria and is involved in the mitochondrial respiration. ***A***, ***B***, Mitochondrial fractionation with *Drosophila* larval muscles (***A***) or mammalian cells (***B***). The larval muscle extracts were fractionated to the cytosolic (Cyto) and mitochondrial (Mito) fractions (***A***). The total extracts (Total) were used as control. Neuro-2a cells were homogenized and fractionated according to the sequential steps (***B***). Collected fractions were analyzed by immunoblotting. PAR-1, Marf, ATP5α, MARK4, MFN2, VDAC1, IP3R3, PCNA, and α-Tub were used to validate the subcellular fractions. PM, plasma membrane fraction. ***C–D***, Colocalization analysis of red-colored PLA dots and GFP-positive subcellular organelles using mitochondria-targeted mitoGFP (***C***) and ER-targeted GFP (GFP-ER; ***D***) in C2C12 cells. Along the dotted yellow arrow within the magnified image (bottom), the gray values of green and red signals were analyzed and plotted using ImageJ software. Scale bar, 10 μm. ***E***, Neuro-2a cells were transfected with MFN2-FLAG and/or MARK4 siRNA (100 nm) as indicated, and then OCRs were measured using a XFe96 extracellular flux analyzer during sequential injections with Oligomycin (1), FCCP (2), and rotenone/antimycin A (3). Error bars indicate SEM. ***F***, Basal respiratory, ATP production, maximal respiratory, and spare respiratory capacity as shown in ***E*** were statistically analyzed. Error bars indicate SE. Statistical significance was determined by a one-way ANOVA test; *p < 0.05, **p < 0.01; n.s. indicates not significant. ***G***, At 48 h after transfection of MFN2-FLAG and/or MARK4 siRNA (100 nm), Neuro-2a cells were harvested and analyzed by immunoblotting with indicated antibodies to check the protein levels.

Next, we addressed whether MARK4/PAR-1 is localized in the mitochondria with MFN2/Marf. We performed a mitochondrial fractionation assay using larval muscle extract, in which the cytosolic and mitochondrial fractions were confirmed by the marker proteins tubulin and ATP5α, respectively. Endogenous PAR-1 was detected in the mitochondrial fraction with Marf, and overexpressed PAR-1-MYC was also present in the mitochondrial fraction of the larval muscle extract (Fig. 4*A*). In addition, MARK4, a conserved homolog of PAR-1, was also isolated in the mitochondrial fraction of Neuro-2a cells together with MFN2, the purity of which was confirmed by the presence of the mitochondrial marker VDAC1 (Fig. 4*B*). However, in the ER fraction, MARK4 was also enriched as previously reported, and MFN2 was found as well consistently with its role in tethering ER and mitochondria. To define the subcellular site of MFN2 and MARK4 physical interaction, we checked whether the MARK4-MFN2 complex is associated with the mitochondria or ER using GFP-tagged organelle markers and PLA signals of MARK4-MFN2 interaction. As Neuro-2a cells, because of their small size, are not appropriate for subcellular analysis, we used C2C12 cells with the larger size to demonstrate intracellular localization more clearly. The PLA signals of MARK4-MFN2 interaction overlapped with the GFP-mitochondria signals (Fig. 4*C*) but not with the GFP-ER signals (Fig. 4*D*), suggesting that MARK4 interacts with MFN2 mainly on the mitochondria.

Previous research has revealed that MFN2 is critical for the mitochondrial respiratory function (Tur et al., 2020). Thus, we further analyzed whether MFN2 and MARK4 interaction affects the mitochondrial respiration as well as morphologic defects. We measured the OCR in Neuro-2a cells transfected with *MFN2-FLAG* or *MARK4* siRNA (Fig. 4*E*). MFN2 overexpression reduced overall OCR values and significantly disrupted the basal, ATP-coupled, maximal, and spare respiratory capacity of the cells compared with the control (Fig. 4*E*,*F*, blue). MARK4 knockdown resulted in an increase in mitochondrial respiratory activity compared with the control (Fig. 4*E*,*F*, green). Interestingly, MARK4 knockdown largely rescued the respiratory abnormalities induced by MFN2 (Fig. 4*E*,*F*, red), but it did not alter protein levels of MFN2-FLAG (Fig. 4*G*). In conclusion, these data indicate that MARK4/PAR-1 cooperates with MFN2/Marf to regulate the respiratory function of mitochondria.

## Discussion

Mitochondrial fusion is crucial for energy production and the maintenance of functional homeostasis, which is governed by the dynamin-like GTPases, Mitofusins. Although dysregulation of mitochondrial fusion proteins has been observed in several human neurodegenerative diseases, the underlying mechanisms have not been fully defined. In this study, we attempted to understand how MFN2/Marf-mediated mitochondrial fusion is regulated and how it is linked to neurodegeneration in *Drosophila* and mammalian cell culture. In *Drosophila*, the *Marf* mutant was reported to have defects in synaptic morphology and transmission by Sandoval et al. (2014), which are regulated nonautonomously by steroid hormones. In their study, knockdown of Marf in motor neurons and muscles does not alter bouton number or size at NMJs. Our analysis also found normal NMJ morphology in presynaptic or postsynaptic knockdown of Marf. Interestingly, we discovered that muscle-specific overexpression of Marf causes the reduction of synaptic boutons, and these defects are synergistically enhanced when combined with PAR-1 overexpression. PAR-1 is known as mainly localized to the postsynaptic region. The postsynaptic overexpression of PAR-1 affects the synaptic bouton number, whereas the presynaptic overexpression does not alter the synaptic structure (Kang et al., 2020). Consistent with the role of PAR-1 in the postsynaptic region, the effect of Marf overexpression mediated by the interaction with PAR-1 was specifically observed in the postsynaptic region. Moreover, the synaptic defects induced by Marf overexpression were significantly restored by PAR-1 downregulation. Given the intrinsic activity of Marf in mitochondrial fusion, our data suggest that the interaction of Marf and PAR-1 links the mitochondrial abnormality to neurodegeneration of the synaptic structure.

Neuroblast-specific PAR-1 overexpression in *Drosophila* has been reported to cause mitochondrial enlargement (Lee et al., 2018). We also observed enlarged mitochondria in the larval muscle and adult wing muscle of PAR-1-overexpressing flies (Extended Data Fig. 3-1), similar to Marf overexpression. Intriguingly, the downregulation of PAR-1 fully restored the hyperfused mitochondrial phenotype induced by Marf overexpression; however, there were no significant changes in mitochondrial morphology when PAR-1 was downregulated alone. These data suggest that PAR-1 modulates Marf-mediated mitochondrial fusion, although the mechanism by which PAR-1 plays a critical role in mitochondrial dynamics remains unknown.

Mitochondrial dynamics are critical for oxidative phosphorylation (OXPHOS) activity (Mishra and Chan, 2016). In this study, we confirmed that MFN2 overexpression causes a reduction in OXPHOS activity in Neuro-2a cells, which has been validated previously (Yu et al., 2019). We also found that the knockdown of MARK4 significantly restored MFN2-induced mitochondrial respiration. Because mitochondria produce ATP through OXPHOS, which is essential for maintaining a healthy neuronal milieu, defects in mitochondrial respiration have frequently been found in many cases of neurodegeneration (Area-Gomez et al., 2019). Therefore, our data suggest that the interaction between MFN2/Marf and MARK4/PAR-1 is implicated in mitochondrial fusion and respiratory activity, contributing to neurodegeneration.

MFN2 localizes not only at the outer mitochondrial membrane but also at the ER and facilitates ER-mitochondria tethering via homotypic (MFN2-MFN2) or heterotypic (MFN1-MFN2) interactions (de Brito and Scorrano, 2008). In our study, MARK4 was detected in the ER-enriched fraction of Neuro-2a cells as well as in the mitochondrial fraction, raising the possibility of MARK4 localization at the mitochondria-associated ER membrane (MAM). Because cellular calcium buffering is a primary role of the MAM, which is critical for neuronal survival and function, it is tempting to speculate that the interaction of MARK4/PAR-1 and MFN2/Marf in the MAM affects calcium homeostasis, the dysregulation of which contributes to neurodegeneration. This hypothesis should be addressed in the future; however, it has been supported by a previous report that calcium homeostasis is relevant to MARK4/PAR-1-induced neurodegeneration (Lee et al., 2018). Although the molecular mechanism underlying the intimate relationship between MARK4/PAR-1 and MFN2/Marf needs to be clarified in the future, our study provides new insights into the link between mitochondrial defects and neurodegeneration, which may offer novel targets for future therapeutic development.

## Synthesis

Reviewing Editor: Niraj Desai, National Institute of Neurological Disorders and Stroke Decisions are customarily a result of the Reviewing Editor and the peer reviewers coming together and discussing their recommendations until a consensus is reached. When revisions are invited, a fact-based synthesis statement explaining their decision and outlining what is needed to prepare a revision will be listed below. The following reviewer(s) agreed to reveal their identity: Hongying Shen. Note: If this manuscript was transferred from JNeurosci and a decision was made to accept the manuscript without peer review, a brief statement to this effect will instead be what is listed below.

One reviewer had an additional comment pertaining to how gene names are written (see below). Please address this comment. Also, a visual abstract would be very useful here.

ADDITIONAL COMMENT

Cheon et al. reported an interaction between MFN2/Marf and MARK4/PAR-1 and their involvement in synaptic defects and mitochondrial dysfunction. MFN2/Marf has been well demonstrated to be involved in mitochondrial function and MARK4/PAR-1 has been reported to be involved in synaptic functions. The causal link between the two genes/proteins is strong and their relationship to neurodegeneration is quite interesting. This manuscript has improved a lot from the previous version submitted to the Journal of Neuroscience. However, the gene names in this manuscript are still quite confusing. Please make them consistent throughout the whole manuscript.

## Author Response

Point-by-point responses to the reviewers

We would like to thank the reviewer for constructive comments on our manuscript. We hope that our responses are clear and to the point.

Reviewer #1: Overall, the findings on the retrograde signaling leading to synaptic degeneration that is induced by enhanced postsynaptic mitochondrial fusion is interesting. The physical and genetic interaction between Marf and PAR-1 is surprising and needs to be explored further.

Cheon et al. presented an interesting finding that impaired mitochondrial dynamics via over-expressing mitofusin (Marf) in the muscle on the postsynaptic site triggered retrograde signaling and degeneration of the synaptic boutons at the NMJ in flies. The phenotype can be enhanced by overexpressing a kinase PAR-1 or rescued by PAR-1 knockdown in the muscle, highlighting an unexpected genetic interaction between Marf and PAR-1. Using biochemistry and cell biology, the authors showed a physical interaction between MFN and mammalian PAR-1 ortholog MARK4, as well as the genetic interaction where MARK4 knockdown suppressed MFN2-overexpression-induced mitochondrial dynamics and respiration defects. Overall, the findings on the retrograde signaling leading to synaptic degeneration that is induced by enhanced postsynaptic mitochondrial fusion is interesting. The physical and genetic interaction between Marf and PAR-1 is surprising and needs to be explored further.

MAJOR COMMENTS:

1) As an important and new finding, the site-of-action supporting Marf and PAR-1 physical interaction needs to be strengthened, whether on the ER, MAM or OMM. As pointing out in the discussion on the role of MFN2 in tethering ER and mitochondria (thus in mediating mitochondria dynamics in fusion and fission) and ER-localization of MARK4 in the Neuro-2a cells, MARK4 localization and site of physical interaction need to be corroborated with new data, i.e., imaging data. Along the same lines, ER markers need to be included as the control in the mitochondrial fractionation experiment for PAR-1/MARK4 shown in Fig. 4A and B.

Thank you for your insightful comments. We carried out several control experiments as you suggested. We included ER marker, IP3R3, in the fractionation experiment and found MFN2 and MARK4 in the ER fraction as well as in the mitochondria fraction (Fig. 4B).

Fig. 4B. MARK4 localizes at the mitochondria and is involved in the mitochondrial respiration. (B) Mitochondrial fractionation with mammalian cells.

We further analyzed the site of MFN2 and MARK4 physical interaction using proximity ligation assay (PLA). The PLA signals of MFN2 and MARK4 associations overlapped with GFP tagged mitochondria maker, but rarely with GFP-tagged ER marker, indicating that MARK4 interacts with MFN2 mainly on the mitochondria. We have added these results in Fig. 4C and 4D and modified on page 6-7 in revised manuscript.

Fig. 4C-D. Co-localization analysis of red-colored PLA dots and GFP-positive subcellular organelles using mitochondria-targeted mitoGFP (C) and ER-targeted GFP (GFP-ER) (D) in C2C12 cells. Along a dotted yellow arrow within the magnified image, the gray values of green and red signals were analyzed and plotted using ImageJ software. Scale bar, 10μm.

2) As another interesting finding, the molecular mechanisms supporting Marf/PAR-1 mediated retrograde signaling leading to synaptic degeneration needs to be explored further. For instance, is PAR-1 kinase activity required for the regulation? Does marf acts upstream or downstream of PAR-1? What’s their direct downstream effectors on the postsynaptic side, for instance, if Dlg or tau phosphorylation (Zhang 2007 and Iijima-Ando 2012), or mitochondrial calcium homeostasis involved in this retrograde signaling? Figure 5 is thus an oversimplified graph and require further experiments in pinpointing the mechanism.

Thank you for pointing out this important issue. We have performed several experiments as the reviewer suggested. We have found that neither the overexpression or knockdown of Marf leads to delocalization of Dlg (Fig.R1). As for the putative signaling by which marf knockdown decreases active PAR-1, we are currently investigating several potential mechanisms as described in the discussion section. We hope to report these studies in the near future, which will adequately respond to the reviewer’s comment. We have now removed Fig. 5 and and carefully revised discussion section.

Fig. R1. Representative images of larval muscle 6/7 in segment A3 immunostained with an anti-Dlg antibody in red and anti-HRP in green. The genotypes are as follows: Mhc-GAL4/+, UAS-Marf-Flag/+; Mhc-GAL4/+ or UAS-Marf RNAi/+; Mhc-GAL4/+.

MINOR COMMENTS:

1) WB or immunostaining need to be shown to validate the overexpression and knockdown for PAR1 and Marf in the epistasis analysis.

In the work of Zhang et. al (2007), we have validated the overexpression and knockdown for PAR-1 transgenic lines (Fig.R2). We also show the validation of the overexpression and knockdown for Marf (Fig. R3).

Fig. R2. Western blot analysis comparing levels of PAR-1 (Zhang et al., 2007)

Fig. R3. Western blot analysis of Marf expression level. (A) Western blot analysis of Marf in Mhc-GAL4>UAS-Marf-flag. (B) Western blot analysis of Marf in Mhc-GAL4>UAS-Marf RNAi. Actin serves as a loading control.

(2) The perinuclear mitochondrial clustering upon MFN2 overexpressing (Fig. 3L) is too striking given the very mild reparation defect (Fig. 4C). The basal respiration curve in Fig 4C is unstable and needs to be repeated to generate more accurate estimation for the basal respiration.

This is an important point, and we thank the reviewer for bringing it up. We suspect that the relatively mild effect of MFN2 overexpression on cellular respiration may be caused by low transfection efficiency. Due to the limitation of bulk cell samples in the OCR measurement assay, the respiration defect in MFN2 overexpressing cells can be masked by the normal cells. The OCR data of MFN2 overexpression in Neuro-2a cells is consistent with the previous data for doxycycline-inducible expression of Mfn2 in KPC cells (Yu et al., 2019). The overall OCR results including basal respiration curves show similar patterns. (Fig. R4)

Fig. R4. Reduced OCR with Mfn2 overexpression and quantified parameters (Yu et al., 2019).

Reviewer #2. The causal link between the two genes/proteins is strong. However, this manuscript suffers from a major issue of not exploring how mitochondria dysfunction affects synaptic defects.

Cheon et al. reported an interaction between MFN2/Marf and MARK4/PAR-1 and their involvement in synaptic defects and mitochondrial dysfunction. MFN2/Marf has been well demonstrated to be involved in mitochondrial function and MARK4/PAR-1 has been reported to be involved in synaptic functions. The causal link between the two genes/proteins is strong. However, this manuscript suffers from a major issue of lack of exploring how mitochondria dysfunction affects synaptic defects. The data presented is fragmented. This manuscript also suffers from the following issues:

MAJOR COMMENTS:

1) Figure 1 shows post-synaptic overexpression of Marf decreases synaptic bouton. In figure S1, pre-synaptic overexpression of Marf shows no effect on the synaptic bouton number. It is necessary to discuss more about why only postsynaptic overexpression of Marf in muscle decreases the synaptic bouton number.

Reviewer’s valuable comment helped us with our research. As the reviewer suggested, we have updated in the revised manuscript. The discussion section on page 8 is amended as follows: “ PAR-1 is known as mainly localized to the postsynaptic region. The postsynaptic overexpression of PAR-1 affects the synaptic bouton number, whereas the presynaptic overexpression does not alter the synaptic structure (Kang et al., 2020) Consistent with the role of PAR-1 in the postsynaptic region, the effect of Marf overexpression mediated by the interaction with PAR-1 was specifically observed in the postsynaptic region”

2) I found Figure 3L-3M was a bit confusing. Is MFN2 a mitochondria marker? Please add a control MFN2 overexpressed with DsRed-Mito to see how it affects the DsRed-Mito distribution. And also add one more group: MFN2 + DsRed-Mito + siMARK4. I think there is a risk to making this current conclusion before adding these two groups.

We regret the lack of clarity. The manuscript has been updated to include the control image of MFN2-YFP and DsRed-Mito overexpression in Extended Data Fig. 3-1. MFN2-YFP and DsRed-Mito colocalized in a clustered pattern, indicating that MFN2 overexpression induces perinuclear clusting of mitochondria marked by DsRed-Mito.

Extended Data Fig 3-1

Extended Data Fig. 3-1. (H) MFN2-YFP colocalizes with DsRed-Mito in a clustered pattern. Representative images of Neuro-2a cells expressing MFN2-YFP (green) and DsRed2-Mito (red). DAPI is shown in blue and the merge in yellow. Scale bar, 10μm.

3) More experiments are required to make this conclusion: “MARK4/PAR-1 is localized to the mitochondria”. The current data from Fig 4A&B indicating that PAR-1 is at the same fraction with mitochondria, is not sufficient to demonstrate its location. The authors could co-stain the endogenous MARK4 with mitochondria marker or at least overexpress MARK4 to evaluate the location.

We agree with the reviewer and appreciate the helpful comments. To clarify our results, we have performed the immunohistochemistry experiments in larvae muscle of the Mhc-GAL4>UAS-mito-GFP. We found that PAR-1 co-localized with mito-GFP in larvae muscle (Fig. R5).

Fig. R5. (A-C) PAR-1 colocalizes with mito-GFP. Representative images of larval muscle 6/7 in segment A3 immunostained with an anti-GFP antibody in green (A), anti-PAR-1 in red (B). The merged image is shown in yellow (C). Scale bar, 10μm.

In addition, to define subcellular site of MFN2 and MARK4 physical interaction in mitochondria, we checked whether the MARK4-MFN2 complex is associated with the mitochondria or ER using GFP-tagged organelle markers and proximity labeling assay (PLA) signals of MARK4-MFN2 interaction. Since Neuro-2a cells, because of their small size, are not appropriate for subcellular analysis, we used C2C12 cells with the larger size to demonstrate intracellular localization more clearly. The PLA signals of MARK4-MFN2 interaction overlap with the GFP-mitochondria signals (Fig. 4C) but not with the GFP-ER signals (Fig. 4D), suggesting that MARK4 interacts with MFN2 mainly on the mitochondria.

Fig. 4C-D. Co-localization analysis of red-colored PLA dots and GFP-positive subcellular organelles using mitochondria-targeted mitoGFP (C) and ER-targeted GFP (GFP-ER) (D) in C2C12 cells. Along a dotted yellow arrow within the magnified image, the gray values of green and red signals were analyzed and plotted using ImageJ software. Scale bar, 10μm.

4) The evidence for genetic interaction between MFN2/Marf and MARK4/PAR-1 is strong. Even there are co-IP results showing the interaction. But still, there is no evidence showing the direct interaction. It would be much better with purified protein binding experiments.

Thank you for the helpful comment. We only suggested the physical association of Marf and PAR-1, not the direct contact. We have also included the PLA experimental evidence of Marf and PAR-1 physical association (Fig. 2C, 4C and 4D). In addition, we have changed “the physical contact” to “physical association” or “interaction” on pages 4-5 in in revised manuscript.

Fig. 2C. Protein-protein interaction by in situ proximity ligation assay (PLA). In situ PLA was performed on fixed Neuro-2a cells with Duolink PLA technology. After incubation with rabbit anti-MARK4 and/or mouse anti-MFN2 antibodies for 2 h, the cells were treated with PLA probes for 1 h and mounted with a mounting solution containing DAPI. Scale bar, 10μm.

Fig. 4C-D. Co-localization analysis of red-colored PLA dots and GFP-positive subcellular organelles using mitochondria-targeted mitoGFP (C) and ER-targeted GFP (GFP-ER) (D) in C2C12 cells. Along a dotted yellow arrow within the magnified image, the gray values of green and red signals were analyzed and plotted using ImageJ software. Scale bar, 10μm.

5) For comparison between more than two groups, One-Way ANOVA or Two-Way ANOVA should be used.

Thank you for your kind suggestions on our manuscript. The manuscript has been updated to indicate the statistical analysis more clearly in the figure legend. In the materials and methods section on page 16, we have also added “Comparisons of different groups in the mean values of multiple datasets were performed using one-way ANOVA with Tukey’s multiple-comparison test”

References

Kang HY, Kim HJ, Kim K, Oh SI, Yoon S, Kim J, Park S, Cheon Y, Her S, Lee M, Lu B, Lee S (2020) Actin-microtubule crosslinker Pod-1 tunes PAR-1 signaling to control synaptic development and tau-mediated synaptic toxicity. Neurobiol Aging 90:93-98.

Yu M, Nguyen ND, Huang Y, Lin D, Fujimoto TN, Molkentine JM, Deorukhkar A, Kang Y, San Lucas FA, Fernandes CJ, Koay EJ, Gupta S, Ying H, Koong AC, Herman JM, Fleming JB, Maitra A, Taniguchi CM (2019) Mitochondrial fusion exploits a therapeutic vulnerability of pancreatic cancer. JCI 5 (16) e126915.

Zhang Y, Guo H, Kwan H, Wang JW, Kosek J, Lu B (2007) PAR-1 kinase phosphorylates Dlg and regulates its postsynaptic targeting at the Drosophila neuromuscular junction.. Neuron 53: 201-215

## References

[B1] Ando K, Maruko-Otake A, Ohtake Y, Hayashishita M, Sekiya M, Iijima KM (2016) Stabilization of microtubule-unbound tau via tau phosphorylation at Ser262/356 by Par-1/MARK contributes to augmentation of AD-related phosphorylation and Aβ42-induced tau toxicity. PLoS Genet 12:e1005917. 10.1371/journal.pgen.1005917 27023670 PMC4811436

[B2] Area-Gomez E, Guardia-Laguarta C, Schon EA, Przedborski S (2019) Mitochondria, OxPhos, and neurodegeneration: cells are not just running out of gas. J Clin Invest 129:34–45. 10.1172/JCI120848 30601141 PMC6307938

[B3] Bocca C, Kane MS, Veyrat-Durebex C, Chupin S, Alban J, Kouassi Nzoughet J, Le Mao M, Chao de la Barca JM, Amati-Bonneau P, Bonneau D, Procaccio V, Lenaers G, Simard G, Chevrollier A, Reynier P (2018) The metabolomic bioenergetic signature of opa1-disrupted mouse embryonic fibroblasts highlights aspartate deficiency. Sci Rep 8:11528. 10.1038/s41598-018-29972-9 30068998 PMC6070520

[B4] Chen CL, Hu Y, Udeshi ND, Lau TY, Wirtz-Peitz F, He L, Ting AY, Carr SA, Perrimon N (2015) Proteomic mapping in live *Drosophila* tissues using an engineered ascorbate peroxidase. Proc Natl Acad Sci U S A 112:12093–12098. 10.1073/pnas.1515623112 26362788 PMC4593093

[B5] Chen H, Detmer SA, Ewald AJ, Griffin EE, Fraser SE, Chan DC (2003) Mitofusins Mfn1 and Mfn2 coordinately regulate mitochondrial fusion and are essential for embryonic development. J Cell Biol 160:189–200. 10.1083/jcb.200211046 12527753 PMC2172648

[B6] Cheon Y, Yoo A, Seo H, Yun SY, Lee H, Lim H, Kim Y, Che L, Lee S (2021) Na/K-ATPase beta1-subunit associates with neuronal growth regulator 1 (NEGR1) to participate in intercellular interactions. BMB Rep 54:164–169. 10.5483/BMBRep.2021.54.3.116 32958118 PMC8016658

[B7] Corrado M, Scorrano L, Campello S (2012) Mitochondrial dynamics in cancer and neurodegenerative and neuroinflammatory diseases. Int J Cell Biol 2012:729290. 10.1155/2012/729290 22792111 PMC3391904

[B8] de Brito OM, Scorrano L (2008) Mitofusin 2 tethers endoplasmic reticulum to mitochondria. Nature 456:605–610. 10.1038/nature07534 19052620

[B9] Iapadre G, Morana G, Vari MS, Pinto F, Lanteri P, Tessa A, Santorelli FM, Striano P, Verrotti A (2018) A novel homozygous MFN2 mutation associated with severe and atypical CMT2 phenotype. Eur J Paediatr Neurol 22:563–567. 10.1016/j.ejpn.2017.12.020 29361379

[B10] Ishikawa K, Yamamoto S, Hattori S, Nishimura N, Tani H, Mito T, Matsumoto H, Miyakawa T, Nakada K (2019) Acquired expression of mutant mitofusin 2 causes progressive neurodegeneration and abnormal behavior. J Neurosci 39:1588–1604. 10.1523/JNEUROSCI.2139-18.2018 30606759 PMC6391572

[B11] Jenkins SM, Johnson GV (2000) Microtubule/MAP-affinity regulating kinase (MARK) is activated by phenylarsine oxide in situ and phosphorylates tau within its microtubule-binding domain. J Neurochem 74:1463–1468. 10.1046/j.1471-4159.2000.0741463.x 10737602

[B12] Jiang S, Nandy P, Wang W, Ma X, Hsia J, Wang C, Wang Z, Niu M, Siedlak SL, Torres S, Fujioka H, Xu Y, Lee HG, Penrry G, Liu J, Zhu X (2018) Mfn2 ablation causes an oxidative stress response and eventual neuronal death in the hippocampus and cortex. Mol Neurodegener 13:5. 29391029 10.1186/s13024-018-0238-8 PMC5796581

[B13] Kang HY, Kim HJ, Kim K, Oh SI, Yoon S, Kim J, Park S, Cheon Y, Her S, Lee M, Lu B, Lee S (2020) Actin-microtubule crosslinker Pod-1 tunes PAR-1 signaling to control synaptic development and tau-mediated synaptic toxicity. Neurobiol Aging 90:93–98. 10.1016/j.neurobiolaging.2020.02.005 32169355 PMC7358005

[B14] Khalil B, Cabirol-Pol MJ, Miguel L, Whitworth AJ, Lecourtois M, Liévens JC (2017) Enhancing Mitofusin/Marf ameliorates neuromuscular dysfunction in *Drosophila* models of TDP-43 proteinopathies. Neurobiol Aging 54:71–83. 10.1016/j.neurobiolaging.2017.02.016 28324764

[B15] Kitay BM, McCormack R, Wang Y, Tsoulfas P, Zhai RG (2013) Mislocalization of neuronal mitochondria reveals regulation of Wallerian degeneration and NMNAT/WLD(S)-mediated axon protection independent of axonal mitochondria. Hum Mol Genet 22:1601–1614. 10.1093/hmg/ddt009 23314018 PMC3657477

[B16] Kuhns S, Schmidt KN, Reymann J, Gilbert DF, Neuner A, Hub B, Carvalho R, Wiedemann P, Zentgraf H, Erfle H, Klingmüller U, Boutros M, Pereira G (2013) The microtubule affinity regulating kinase MARK4 promotes axoneme extension during early ciliogenesis. J Cell Biol 200:505–522. 10.1083/jcb.201206013 23400999 PMC3575539

[B17] Leal NS, Schreiner B, Pinho CM, Filadi R, Wiehager B, Karlström H, Pizzo P, Ankarcrona M (2016) Mitofusin-2 knockdown increases ER-mitochondria contact and decreases amyloid β-peptide production. J Cell Mol Med 20:1686–1695. 10.1111/jcmm.12863 27203684 PMC4988279

[B18] Lee H, Smith SB, Yoon Y (2017) The short variant of the mitochondrial dynamin OPA1 maintains mitochondrial energetics and cristae structure. J Biol Chem 292:7115–7130. 10.1074/jbc.M116.762567 28298442 PMC5409478

[B19] Lee KS, Huh S, Lee S, Wu Z, Kim AK, Kang HY, Lu B (2018) Altered ER-mitochondria contact impacts mitochondria calcium homeostasis and contributes to neurodegeneration *in vivo* in disease models. Proc Natl Acad Sci U S A 115:E8844–e8853. 30185553 10.1073/pnas.1721136115 PMC6156612

[B20] Lee S, Wang JW, Yu W, Lu B (2012) Phospho-dependent ubiquitination and degradation of PAR-1 regulates synaptic morphology and tau-mediated Aβ toxicity in *Drosophila*. Nat Commun 3:1312. 10.1038/ncomms2278 23271647 PMC4307937

[B21] Mishra P, Chan DC (2016) Metabolic regulation of mitochondrial dynamics. J Cell Biol 212:379–387. 10.1083/jcb.201511036 26858267 PMC4754720

[B22] Mishra P, Carelli V, Manfredi G, Chan DC (2014) Proteolytic cleavage of Opa1 stimulates mitochondrial inner membrane fusion and couples fusion to oxidative phosphorylation. Cell Metab 19:630–641. 10.1016/j.cmet.2014.03.011 24703695 PMC4018240

[B23] Naz F, Anjum F, Islam A, Ahmad F, Hassan MI (2013) Microtubule affinity-regulating kinase 4: structure, function, and regulation. Cell Biochem Biophys 67:485–499. 10.1007/s12013-013-9550-7 23471664

[B24] Nishimura I, Yang Y, Lu B (2004) PAR-1 kinase plays an initiator role in a temporally ordered phosphorylation process that confers tau toxicity in *Drosophila*. Cell 116:671–682. 10.1016/s0092-8674(04)00170-9 15006350

[B25] O'Rourke B (2016) Metabolism: beyond the power of mitochondria. Nat Rev Cardiol 13:386–388. 10.1038/nrcardio.2016.95 27303815

[B26] Saito T, Oba T, Shimizu S, Asada A, Iijima KM, Ando K (2019) Cdk5 increases MARK4 activity and augments pathological tau accumulation and toxicity through tau phosphorylation at Ser262. Hum Mol Genet 28:3062–3071. 10.1093/hmg/ddz120 31174206

[B27] Sandoval H, Yao CK, Chen K, Jaiswal M, Donti T, Lin YQ, Bayat V, Xiong B, Zhang K, David G, Charng WL, Yamamoto S, Duraine L, Graham BH, Bellen HJ (2014) Mitochondrial fusion but not fission regulates larval growth and synaptic development through steroid hormone production. Elife 3:e03558. 10.7554/eLife.03558 25313867 PMC4215535

[B28] Sheridan C, Martin SJ (2010) Mitochondrial fission/fusion dynamics and apoptosis. Mitochondrion 10:640–648. 10.1016/j.mito.2010.08.005 20727425

[B29] Tilokani L, Nagashima S, Paupe V, Prudent J (2018) Mitochondrial dynamics: overview of molecular mechanisms. Essays Biochem 62:341–360. 10.1042/EBC20170104 30030364 PMC6056715

[B30] Tomancak P, Piano F, Riechmann V, Gunsalus KC, Kemphues KJ, Ephrussi A (2000) A *Drosophila melanogaster* homologue of *Caenorhabditis elegans* par-1 acts at an early step in embryonic-axis formation. Nat Cell Biol 2:458–460. 10.1038/35017101 10878812

[B31] Trinczek B, Brajenovic M, Ebneth A, Drewes G (2004) MARK4 is a novel microtubule-associated proteins/microtubule affinity-regulating kinase that binds to the cellular microtubule network and to centrosomes. J Biol Chem 279:5915–5923. 10.1074/jbc.M304528200 14594945

[B32] Tur J, Pereira-Lopes S, Vico T, Marín EA, Muñoz JP, Hernández-Alvarez M, Cardona PJ, Zorzano A, Lloberas J, Celada A (2020) Mitofusin 2 in macrophages links mitochondrial ROS production, cytokine release, phagocytosis, autophagy, and bactericidal activity. Cell Rep 32:108079. 10.1016/j.celrep.2020.108079 32846136

[B33] Wang ZH, Clark C, Geisbrecht ER (2016) Analysis of mitochondrial structure and function in the *Drosophila* larval musculature. Mitochondrion 26:33–42. 10.1016/j.mito.2015.11.005 26611999 PMC4752903

[B34] Wieckowski MR, Giorgi C, Lebiedzinska M, Duszynski J, Pinton P (2009) Isolation of mitochondria-associated membranes and mitochondria from animal tissues and cells. Nat Protoc 4:1582–1590. 10.1038/nprot.2009.151 19816421

[B35] Wu Y, Griffin EE (2017) Regulation of cell polarity by PAR-1/MARK Kinase. Curr Top Dev Biol 123:365–397. 28236972 10.1016/bs.ctdb.2016.11.001 PMC5943083

[B36] Youm TH, Woo S-H, Kwon E-S, Park SS (2019) NADPH oxidase 4 contributes to myoblast fusion and skeletal muscle regeneration. Oxid Med Cell Longev 2019:3585390. 10.1155/2019/3585390 31827673 PMC6885834

[B37] Yu M, Nguyen ND, Huang Y, Lin D, Fujimoto TN, Molkentine JM, Deorukhkar A, Kang Y, San Lucas FA, Fernandes CJ, Koay EJ, Gupta S, Ying H, Koong AC, Herman JM, Fleming JB, Maitra A, Taniguchi CM (2019) Mitochondrial fusion exploits a therapeutic vulnerability of pancreatic cancer. JCI Insight 5:e126915. 31335325 10.1172/jci.insight.126915 PMC6777817

[B38] Zhang Y, Guo H, Kwan H, Wang JW, Kosek J, Lu B (2007) PAR-1 kinase phosphorylates Dlg and regulates its postsynaptic targeting at the *Drosophila* neuromuscular junction. Neuron 53:201–215. 10.1016/j.neuron.2006.12.016 17224403 PMC1855201

[B39] Ziviani E, Tao RN, Whitworth AJ (2010) *Drosophila* parkin requires PINK1 for mitochondrial translocation and ubiquitinates mitofusin. Proc Natl Acad Sci U S A 107:5018–5023. 10.1073/pnas.0913485107 20194754 PMC2841909

