# Supplementary figures and images for: A Novel Interaction between MFN2/Marf and MARK4/PAR-1 Is Implicated in Synaptic Defects and Mitochondrial Dysfunction
Source: eNeuro. 2023 Aug 14;10(8):ENEURO.0409-22.2023. doi: 10.1523/ENEURO.0409-22.2023 (PMC10444538; doi:10.1523/ENEURO.0409-22.2023)

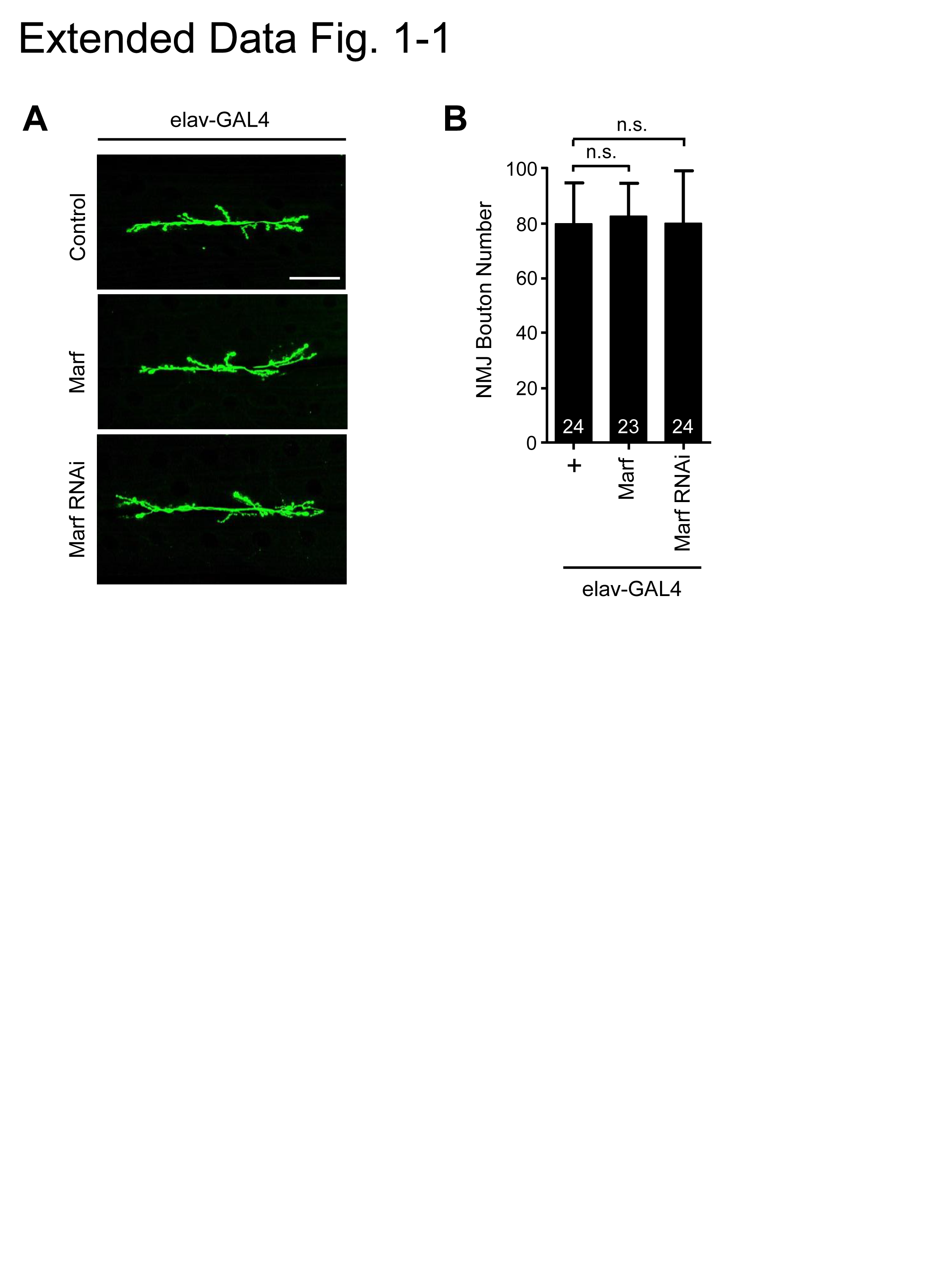

Supplement: Figure 1-1 — Presynaptic-specific overexpression or downregulation of Marf does not affect NMJ bouton number. A, Representative NMJ images of larval muscle 6/7 in segment A3 immunostained with an anti-HRP antibody. The genotypes are as follows: elav-GAL4/+ as control, elav-GAL4>UAS-Marf-FLAG, and elav-GAL4>UAS-Marf RNAi. Scale bar, 50 μm. B, Quantification of data shows that Marf overexpression in presynapse has no effect on the number of boutons. The data are shown as mean ± SD. Statistical significance was determined by one-way ANOVA test; n.s. Download Figure 1-1, TIF file. [file enu-eN-NWR-0409-22-s02.tif]

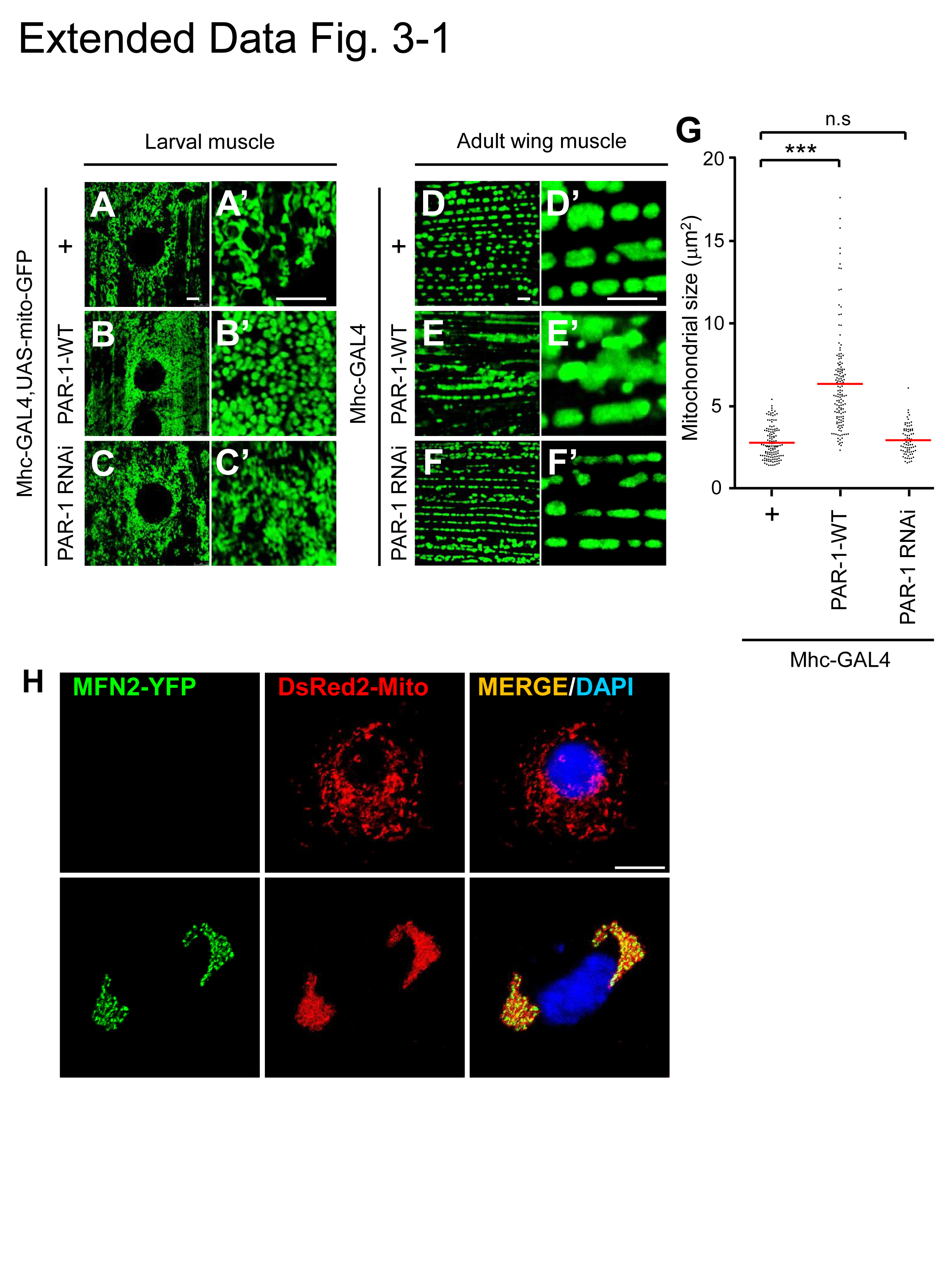

Supplement: Figure 3-1 — Overexpression of PAR-1 in muscles results in mitochondrial hyperfusion. A–F, The mitochondria were visualized in the larval muscle 6/7 of A3 by UAS-mito-GFP (A–C) and in adult wing muscle by immunostaining with anti-ATP5α antibody (D–F). Each image is magnified on the right (A’–F’). Scale bar, 5 μm. The genotypes of larval muscle are Mhc-GAL4>UAS-mito-GFP as a control (A), Mhc-GAL4>UAS-mito-GFP+UAS-PAR-1-WT (B), and Mhc-GAL4>UAS-mito-GFP+UAS-PAR-1 RNAi (C). The genotypes of adult muscle are Mhc-GAL4/+ as a control (D), Mhc-GAL4>UAS-PAR-1-WT (E), and Mhc-GAL4>UAS-PAR-1 RNAi (F). G, Quantification of mitochondrial size in the adult wing muscle. The number of mitochondria analyzed in each genotype is as follows: Mhc-GAL4/+ (n = 133), Mhc-GAL4>UAS-PAR-1-WT (n = 145), and Mhc-GAL4>UAS-PAR-1 RNAi (n = 77). The data are shown as mean ± SD. Statistical significance was determined by a one-way ANOVA test; ***p < 0.001; n.s. H, MFN2-YFP colocalizes with DsRed-Mito in a clustered pattern. Representative images of Neuro-2a cells expressing MFN2-YFP (green) and DsRed2-Mito (red). DAPI is shown in blue, and co-localized regions were indicated as yellow. Scale bar, 10 μm. Download Figure 3-1, TIF file. [file enu-eN-NWR-0409-22-s03.tif]
